# Valorization of mango byproducts for sustainable active packaging: Advances in functionalized biopolymer films

**DOI:** 10.1016/j.fochx.2025.102961

**Published:** 2025-08-26

**Authors:** Guihong Fang, Bangdi Liu, Junyan Guo, Gulden Goksen, Mansuri M. Tosif, Shima Jafarzade, Parya Ezati, Ananthi Pandi

**Affiliations:** a School of Public Health, International Collaborative Research Center for the Development and Utilization of Tropical Food for Special Medical Purpose, Hainan Medical University, Haikou 571199, China; b Academy of Agricultural Planning and Engineering, MARA, Beijing 100125, China; c Key Laboratory of Agro-Products Primary Processing, Beijing 100125, China; d School of Food Science and Engineering, Hainan University, Haikou 570228, China; e Department of Food Technology, Vocational School of Technical Sciences at Mersin Tarsus Organized Industrial Zone, Tarsus University, 33100 Mersin, Turkey; f Centre for Sustainable Bioproducts, Deakin University, Waurn Ponds, VIC 3216, Australia; g Department of Food Science, University of Guelph, ON N1G2W1, Canada; h Department of Chemistry, The Gandhigram Rural Institute – Deemed to be University, Gandhigram, Dindigul 624 302, Tamil Nadu, India

**Keywords:** Mango waste, Phenols, Active packaging, Biopolymer

## Abstract

Faced with the environmental crisis caused by plastic waste, recent research has focused on the development of biopolymer-based food packaging films with enhanced properties, among which agricultural byproducts are promising resources. This review comprehensively analyzes the application value of different mango byproducts—peel, seeds, and leaves—in sustainable active packaging films. It evaluates the latest progress in utilizing these wastes: peel phenolics and pectins can enhance antioxidant, UV-protective, and smart pH-responsive properties; seed starch, kernel extracts, and oils can improve mechanical strength, thermal stability, and hydrophobicity; and leaf bioactives exhibit strong antimicrobial functions. This work innovatively synthesizes strategies for extracting these functional components and integrating them into various biopolymer matrices (e.g., chitosan, polylactic acid, starch), demonstrating their efficacy in significantly extending the shelf life of perishable foods. This study highlights mango byproducts as a versatile and environmentally friendly alternative that aligns with circular economy principles and has the potential to transform waste into high-value packaging materials—despite challenges in standardization and scalability.

## Highlights

• The main by-products of mango include mango peel and mango seeds.
• Mango peel extract can be a multifunctional additive for active packaging.
• Mango kernel extract can be used to develop hydrophobic active food packaging.
• Mango waste can significantly enhance food preservation capabilities of packaging film.

## Graphical abstract

**Figure:**
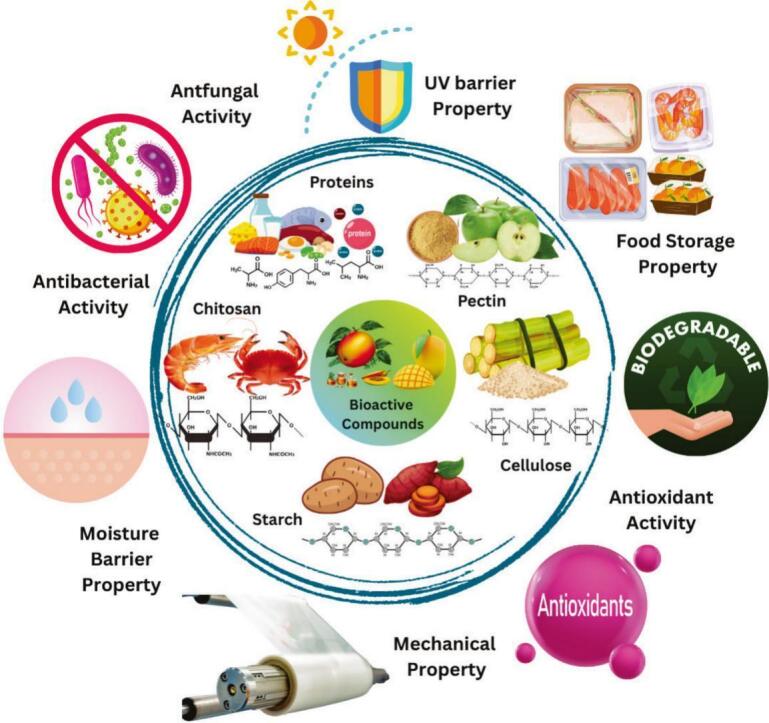

## 1 Introduction

In recent years, the development of sustainable food packaging materials has garnered significant attention due to the growing environmental burden associated with petroleum-based plastics (Chen, Guo, et al., 2024). Conventional plastic packaging, though effective in preserving food quality and extending shelf life, contributes substantially to long-term pollution due to its non-biodegradable nature (Adilah, Jamilah, & Hanani, 2018; García-Mahecha et al., 2023). As a response to these environmental challenges, biopolymer-based food packaging films have emerged as promising alternatives due to their biodegradability, renewability, and compatibility with food systems (Su et al., 2024; Zhang, Azizi-Lalabadi, et al., 2023; Zhang, Roy, Ezati, Yang, & Rhim, 2023; Zhang et al., 2024). At present, compared with traditional petroleum-based plastic food packaging, biopolymer-based food packaging still faces limitations such as insufficient comprehensive performance and high cost. Therefore, many studies have focused on developing biopolymer-based food packaging films with enhanced overall properties. Various functional additives and film-forming strategies are employed to improve the mechanical strength, barrier properties, antibacterial activity, and antioxidant capacity of biopolymer-based food packaging films (Guo, Rawdkuen, Alamri, et al., 2025,Guo, Rawdkuen, Zhang, et al., 2025). Among various innovations in this domain, the incorporation of agro-industrial byproducts into biopolymer matrices has gained considerable momentum as a dual-purpose strategy improving the functional attributes of packaging films while valorizing food processing waste (Mwaurah et al., 2020; Rambabu et al., 2019). Among various agricultural wastes, fruit waste has received extensive attention in recent decades. In particular, wastes such as peels and cores have become excellent sources of many bioactive substances and are widely used in the food industry (Shukla et al., 2024). Over the past decade, fruit waste has been increasingly recognized as a valuable resource for biopolymer-based food packaging films. Different types of fruit waste can be used to produce biopolymers such as pectin and starch, as well as functional additives such as polyphenol extracts and essential oils (Dubey et al., 2023).

Mango (*Mangifera indica* L.), one of the most widely consumed tropical fruits globally, generates a significant quantity of byproducts such as peels, seeds, and kernels during industrial processing (Farouk, 2024; Lebaka et al., 2021). These mango-derived residues are rich sources of bioactive compounds, including polyphenols, flavonoids, dietary fibers, and phytosterols, which possess potent antioxidant, antimicrobial, and anti-inflammatory properties. Traditionally discarded or underutilized, mango byproducts are now being investigated as value-added ingredients in biopolymer-based packaging systems (Adilah, Jamilah, & Hanani, 2018). Their integration into film formulations not only enhances the active functionality of the packaging but also aligns with circular economy principles and sustainable resource utilization (Choudhary et al., 2023). The use of mango peels and seed extracts as natural additives in biopolymer matrices such as starch (ST), chitosan (CS), cellulose, alginate, and polylactic acid (PLA) has shown promising results in recent studies (Jafarzadeh et al., 2020; Oliver-Simancas et al., 2021). These functionalized films exhibit improved barrier properties, mechanical strength, UV protection, and bioactivity, thereby contributing to prolonged food shelf life and improved safety (Vilvert et al., 2023). For instance, mango peel extract is particularly rich in gallic acid, mangiferin, and other phenolic compounds that have demonstrated strong free radical scavenging and antimicrobial activities (Nogueira et al., 2020). Incorporating these compounds into film structures enables the development of active packaging that can respond to internal and external food quality changes (Quintana et al., 2021).

Moreover, mango byproducts also offer excellent film-forming aids due to their high fiber and pectin content, which can improve the structural integrity and flexibility of biopolymer films. Mango kernel fat, an emerging component extracted from mango seeds, is a potential plasticizer alternative that can replace synthetic additives, thereby enhancing film flexibility and water resistance (Manzoor et al., 2022). This multifunctionality positions mango byproducts as both structural and functional enhancers in packaging formulations. The functionalization of food packaging films using mango byproducts has also benefited from green extraction techniques such as ultrasound-assisted extraction, supercritical CO₂ extraction, and microwave-assisted extraction (Susmitha, Sasikumar, Rajan, & Nampoothiri, 2021). These methods ensure high recovery of bioactive compounds with minimal degradation, enabling their effective incorporation into biodegradable films (Ponnusamy et al., 2025a, Ponnusamy et al., 2025b). Additionally, recent advances in nanotechnology have led to the encapsulation of mango seed or peel extracts into nanocarriers, further improving their stability, controlled release, and compatibility with hydrophilic and hydrophobic film matrices (Kučuk et al., 2024). Several studies have reported encouraging outcomes regarding the physicochemical, barrier, and antimicrobial characteristics of mango-byproduct-functionalized films (Kamarudin et al., 2022; Salahuddin et al., 2021). Films based on CS or ST incorporated with mango peel extract demonstrated enhanced oxygen and moisture barrier properties, making them suitable for high-perishability foods like meat and dairy products (Reis et al., 2015). Other reports revealed that mango seed polyphenols, when added to PLA-based films, significantly delayed lipid oxidation and microbial growth in packaged food items. These findings highlight the promising potential of mango byproducts as cost-effective and sustainable functional agents in the development of next-generation food packaging materials (del Pilar Sánchez-Camargo et al., 2021). Furthermore, this research direction addresses multiple Sustainable Development Goals (SDGs), including responsible consumption and production (SDG 12), climate action (SDG 13), and zero hunger (SDG 2). By valorizing agro-industrial waste and minimizing plastic usage, these innovations support the transition to a bio-based circular economy (Elsayed et al., 2022). This approach also enhances the value chain of mango processing industries, offering new revenue streams and contributing to waste reduction. Despite these promising developments, certain challenges persist (Ali et al., 2025). The variability in chemical composition of mango byproducts based on cultivar, geography, and processing methods can lead to inconsistencies in film performance. Additionally, potential sensory impacts such as odor, color, or flavor migration from the films to packaged food must be critically assessed (Manhongo et al., 2021). Standardization of extraction and film preparation protocols, regulatory approval of bioactive components, and scalability of production processes are also crucial factors for commercial translation (Deshmukh & Gaikwad, 2024; Singh et al., 2022).

Therefore, this review aims to integrate the latest research progress in the field of mango by-product-functionalized biopolymer-based food packaging films. It first briefly introduces the characteristics and main components of different mango by-products, focusing on the impact of mango peel, mango seed, mango flesh, and mango leaves on the comprehensive properties of biopolymer-based food packaging films. It also summarizes the application progress of such films in food preservation. Through a comprehensive evaluation, this review seeks to provide valuable insights into the current status and future directions of mango waste valorization in the field of active and intelligent food packaging applications.

## 2 Mango by-products

Mango is one of the most demanding and delicious tropical fruits and is famous due to its rich source of nutrients and bioactive compounds. Furthermore, it is consumed directly as a fresh fruit and in its value-added form, like smoothies, snacks, jams, juices, and frozen fruits (Lebaka et al., 2021; Siddiq et al., 2017). However, processing of mangoes produces a significant number of by-products which are rich in several bioactive compounds. Although mango pulp is widely used for the production of value-added food products, and remaining peel, leaf, and seeds are considered waste material (García-Mahecha et al., 2023). The number of by-products of the mangoes can vary depending on the variety. These by-products (seed and peel) are not only waste material, they are a reservoir of potential bioactive compounds such as dietary fibers, vitamins, oils, and polyphenols, which create opportunities for their valorization and integration in diverse food industrial applications (Okino Delgado & Fleuri, 2016). However, several studies showed that whole by-products can amount to nearly 60 %. Herein, seeds are considered as discarded material, which is rich in ST and potentially used for the preparation of packaging materials. According to data, it has been shown that around 35–40 % of mango by-products are generated from the seed only followed by 15–20 % from peels (Teshome et al., 2023). In the context of extraction methods, the different extraction solvents, including water, acetone, methanol, and ethanol, are widely used to extract the bioactive compounds from mango by-products. Aqueous ethanol extraction is potentially preferred because of its food-grade status and effectively recovers the antioxidant compounds (del Pilar Sánchez-Camargo et al., 2021; García-Mahecha et al., 2023; Lim et al., 2019). Moreover, microwave-assisted extraction and ultrasound-assisted extraction are widely employed to increase the yield and reduce the extraction time. Consequently, different bioactive compounds from the mango peels, seed, and leaves have been encapsulated using carriers like CS, cyclodextrins, maltodextrins, and polysaccharides like CS have been utilized to stabilize the phenolics from mango by-products. Therefore, advanced extraction methods and the incorporation of bioactive compounds into the production of functional foods and packaging materials provide a natural alternative to synthetic material.

### 2.1 Mango peels

**Fig. 1:**
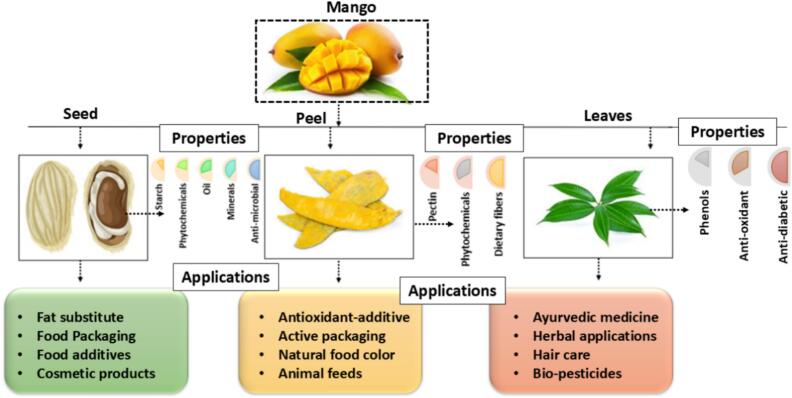
Different by-products of mango and their key composition and properties.

Mango peels are effectively used for the production of different novel food products. Moreover, mango peel and kernel are tropical by-products that are high in dietary fiber due to the existence of an abundance of polysaccharides such as pectin, cellulose, and ST (Kaur et al., 2023; Lebaka et al., 2021). These polysaccharides are in the form of simple sugars, including mannose, galactose, and arabinose. On the other hand, seeds and peels are rich in bioactive compounds, which are helpful for different food applications (Fig. 1). Moreover, mango peels exhibited higher amounts of minerals and essential elements such as iron, phosphorus, magnesium, potassium, and calcium. The amount of these elements can be varied depending on the variety and maturation stages of the mango. Additionally, it has been shown that mango peel contains different vitamins (vitamin A, C and vitamin E) which further increases the biological importance of peels. Whereas, peels exhibited higher anti-oxidant activities compared to mango pulp. Thus, in future, researcher should work on the incorporation of mango peels into value added food product which may benefits for human health. In this content, Ojeda et al. (2023) extracted the phenolic compounds from the mango seeds and peels using deep eutectic solvents. They used agitation and ultrasound methods to study the effect of deep eutectic solvents on the bioactive compounds. Results showed that choline chloride and β-alanine-based solvents significantly extracted the higher polyphenols from the seed and peel. The solvents extracted polyphenols three times higher from peels (23.05 mg/g DW) and five times higher from the seeds (60.01 mg/g DW). On the other hand, these by-products also contain phytochemicals, which are known to be bioactive substances with proven health-promoting properties. Seed and peel contain higher amount of xanthanoids, benxophenones, catechins, flavonoids, gallotannins, and different derivatives of gallic acid. Similarly, mango peel powder was employed as an emulsifying and thickening agent due to presence of the higher amount of pectin for the production of low-fat chicken patties (Chappalwar et al., 2020). In this study, mango peel powder was utilized as a vegetable oil (50 %) substitute and results showed the significant impact on the color characteristics and textural properties. Also, mango peels are effectively used for the replacement of different ingredients like wheat flour and sugar cane. In a study done by Ramirez-Maganda et al. (2015), they replaced 75 % of wheat flour and sugar cane in muffins by enhancing the physicochemical properties, sensory aspects, and bioactive compounds. Likewise, mango by-products have attained great interest as a functional additive to improve the properties of biopolymeric films, by offering a sustainable and economically viable approach to improve food preservation and enhance the shelf-life (Dilucia et al., 2020). In this way, many reports have been published on the effect of mango peel extract for the development of packaging materials. Herein, the addition of mango peel powder extract significantly enhanced the free radical scavenging activities and decreased the solubility and permeability of films (Adilah, Jamilah, Noranizan, & Hanani, 2018).

### 2.2 Mango seed or kernelical

The mango seed is covered with a hard outer shell coating a soft kernel, which makes up nearly 10–25 % of the total fruit weight, depending on the variety. Usually, mango kernel is composed of 6–8 % protein, 11–13 % fat, and 8–10 % ST, along with a minor number of bioactive compounds such as phytosterols and phenolic compounds (How et al., 2024; Mwaurah et al., 2020). Mango kernel is also famous because of a higher amount of fat content; Therefore, it can be referred to as a mango butter, which has a fatty acid composition rich in oleic acids and stearic acids. These properties make it a potential material for the food and cosmetic industries (Jin et al., 2019). To date, most of the research has focused on the extraction of ST from the mango kernel and its utilization in different applications. Also, in a few studies, mango kernel was used as a fat replacer in different foods. For instance, Kumar et al. (2023) utilized the mango kernel powder for the production of low-fat cookies. Results revealed that addition of mango seed kernel significantly improved the physicochemical properties, and biochemical properties of cookies. Therefore, mango seed kernel can be potentially used as a natural and cost-effective ingredient for the replacement of fat. In another study, Kaur et al. (2022) used the mango seed kernel for the substitution of cocoa butter in chocolate. Also, study highlighted the effect of mango seed kernel on the physicochemical characterization n and textural properties of chocolate. Characterization data proved that, mango seed kernel fat successfully replaced the cocoa butter without compromising the properties of chocolate. Also, kernel-based chocolate showed similar composition of stearic acids, oleic acid, and palmitic acid. Overall, study revealed that, mango seed kernel has ability to replace the cocoa butter without compromising the organoleptic properties of chocolate. Consequently, mango seed kernel displayed higher phenolic compounds with biological properties than edible fraction of mango. However, different factors are affected on these parameters such as type of cultivar, geographical location, and maturation degree. In this content, Alañón et al. (2021) worked on the different cultivars (Osteen, Kent, and Keitt) of mangoes at five different maturation stage. The findings showed that during the first three phases of ripening, “Keitt” samples had greater levels of iriflophenone glucoside, maclurin C-glucoside, maclurin digalloyl glucoside, mangiferin, 5-galloyl quinic acid, and trigalloyl glucose. Nonetheless, the “Osteen” variety's seed kernel had greater levels of hexa- and hepta-gallotannins, which concentrations decreased as the seed matured. Thus, maturity of fruits effects on the overall bioactive compounds and nutritional compounds of the material. Moreover, the nutritional composition of plant-derived edible oils has kept mango kernel in the high demand. Unsaturated fatty acids are highly existing in plant-based oils and fats. In this context, Awolu and Manohar (2019), estimated the qualitative and quantitative analysis of mango seed kernel oil, extracted using supercritical CO_2_ and solvent extraction methods. Different solvents such as acetone, ethanol, petroleum ether, and hexane were used to extract the oil. Results indicated that supercritical extraction exhibited lower yield (2.5–3.6 %) compared to solvent extraction (8.02–19.88 %). Whereas, except for the acetone extracted oil, the unsaturated fatty acid (UFA) content of the extracted oil utilizing the solvent extraction approach was greater. Since ethanol extracted oil was characterized as having extraneous components, as shown by the thermal profile, FTIR, and microscopy, it might not be a good solvent for extracting oil from oil seeds. Overall, the mango seed kernel is rich in oil and can be used in diverse industrial applications as a natural, cost-effective ingredient.

Mango by-products' application in packaging applications is further enhanced by sustainability considerations. In accordance with the principles of the circular economy, recognizing mango waste not only solves the problem of agro-industrial disposal but also reduces dependence on artificial antioxidants and preservatives (Oliver-Simancas et al., 2021). In comparison to traditional plastic-based systems, life cycle assessment (LCA) studies on biopolymers made from mango waste have shown decreases in resource use and carbon footprint (Dilucia et al., 2020).

Mango by-products, particularly the peel and seed kernel, have a lot of promise for use as beneficial additives in food packaging films made of biopolymers. Their abundance of bioactive chemicals offers a variety of advantages, including antibacterial, antioxidant, UV-protective, and even sensory-responsive qualities, which can improve the sustainability and performance of packaging materials that are edible and biodegradable (Tirado-Kulieva et al., 2021). With advancements in mechanical, thermal, and barrier properties, an increasing amount of research supports their incorporation into a variety of polymer matrices, such as cellulose derivatives, ST, CS, gelatin, and pectin. In order to migrate mango by-product functionalisation from lab-scale prototypes to commercial packaging solutions, future research should concentrate on consumer-friendly innovations, synergistic interactions with other plant-based bioactives, and scale-up strategies.

### 2.3 Mango leaves

**Fig. 2:**
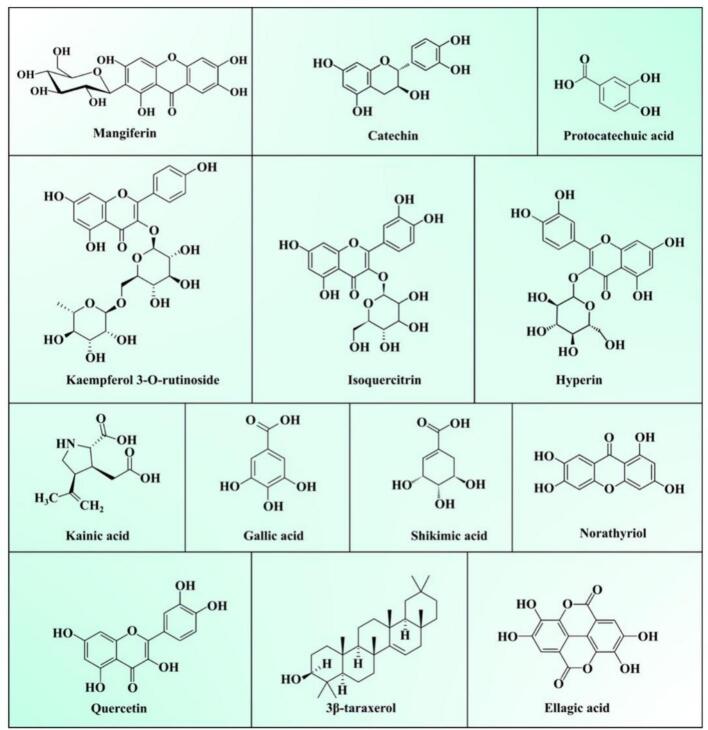
Structure of major compounds present in mango leaves (Kumar et al., 2021).

Mango leaves are used in ayurvedic medicine, and it is increasingly being explored for functional food and industrial applications. Mango leaves are rich in mangiferein, flavonoids, phenolic acids, and xanthone compounds with remarkable therapeutic applications (Mirza et al., 2021). Several studies revealed that mango leaves have higher phenolic compounds than other fruit leaves, which makes them a promising bioactive material for food-grade biopolymers. Similarly, dry mango lead extract showed excellent antimicrobial properties against harmful food pathogenic bacteria, including *Escherichia coli* and *S. auresus*, and can be incorporated into edible or biodegradable films for the shelf-life enhancement of food (Tavassoli-Kafrani et al., 2022). Furthermore, the addition of leaf extract into the different polymers (carboxymethyl cellulose (CMC), gelatin, and PLA improved the physicochemical properties and thermal stability of films (Farouk, 2024; Kupervaser et al., 2023). It is worth noting that the main components of mango leaves from different varieties and growth stages vary. For example, the crude protein content of the Mauritius Dauphiné variety is 13.6 %, the Nigerian variety is 20.38 %, and the Laotian variety is 6.90 % (Jhaumeer Laulloo et al., 2018). In addition, the volatile components of mango leaves also differ among varieties. For instance, the main components of mango leaf oil extracted from the Tommy Atkins variety are β-carene (29.64 %), caryophyllene oxide (12.40 %), and epoxyhumulene II (8.66 %), whereas the primary components of mango leaf oil extracted from the Rosa, Moscatel, and Jasmim varieties are caryophyllene oxide and epoxyhumulene II (D [zbreve] amić et al., 2010). The most valuable components of mango leaves are polyphenols, including phenolic acids, flavonoids, benzophenones, tannins, terpenoids, and flavonoids (Fig. 2) (Kumar et al., 2021). Mangiferin is a potential bioactive compound in mango leaf extract. In recent years, a diet rich in bioactive compounds has attracted much attention due to its ability to minimize the risk of several chronic diseases. Studies have shown that mangiferin (7.43 %) is the major component of mango leaf extract, while other compounds reported in higher concentrations include quercetin-3-O-β-D-glucoside (0.82 %), quercetin-3-O-β-D-galactoside (0.86 %), and isocarpic acid (1.25 %) (Pan et al., 2018). However, as mentioned above, the main components of mango leaves at different growth stages are also different. From the perspective of industrial application, fruiting mango leaves should be selected as waste resources. Therefore, future research should explore the changes in the main components of mango leaves at different growth stages.

In addition to the above mango byproducts, mango tree stems are also a promising byproduct that can be used to prepare food packaging materials. Mango tree stems contain a large amount of cellulose, which can be used to prepare nanocellulose. However, there is no research on the use of mango tree stems to develop food packaging materials. Over the past few decades, mango by-products have been potentially used in food industrial applications. However, they have found limited applications in pharmaceutical applications, animal feed, as a pectin source, and composting. Also, current research and technology are focusing on the utilization of mango by-products in high-value sectors like bio-medical, pharmaceutical, and nutraceuticals. Mango by-products have promise for use in intelligent packaging solutions in addition to their mechanical and antioxidant properties. It is possible to create freshness indicators or spoiling sensors by using the colorimetric responses of the natural pigments found in mango peel, such as carotenoids and polyphenols, to pH variations. The potential for consumer-interactive packaging that instantly conveys quality changes is presented by these applications, albeit they are still in the early stages (Mohd Basri et al., 2021). Despite promising properties and research findings of mango by-products, there are many challenges and drawbacks to the widespread adoption of these materials in biopolymer-based packaging. In this way, a major challenge is to variability in composition across the different varieties and cultivars. Also, the limited scalability of extraction and purification processes are still at the emerging stage, and regulatory hurdles concerning food contact materials. Therefore, future research should focus on the optimization and standardization of extraction protocols with enhanced compatibility of mango by-products with several biopolymer metrics. Also, there is a need for exploration of novel delivery systems (nanoemulsions, nanofibers, and multilayer films).

## 3 Research progress of food packaging films functionalized with mango peel

### 3.1 Mango Peel extract

The incorporation of bioactive compounds from agro-industrial waste into food packaging materials is gaining considerable attention as a sustainable and eco-friendly alternative to conventional synthetic additives. Mango peel extract (MPE), derived from the peels of *Mangifera indica*, has emerged as a promising bioactive agent due to its abundance of polyphenolic compounds, flavonoids, dietary fibers, and potent antioxidants. Mango processing industries generate large quantities of peel as a byproduct, often leading to environmental disposal challenges. Utilizing this waste stream for packaging applications addresses two significant concerns simultaneously: reducing agro-industrial waste and developing functional food packaging materials with enhanced performance.

MPE is particularly valued for its rich phenolic content, including gallic acid, caffeic acid, mangiferin, quercetin, and catechins, which contribute to its strong antioxidant and antimicrobial properties. These bioactivities are essential in food packaging applications where oxidative degradation and microbial contamination are major concerns affecting food shelf life and safety. MPE not only prolongs the shelf life of food products by inhibiting microbial growth but also improves the oxidative stability of the packaged food, thereby maintaining its nutritional quality and sensory attributes. Researchers have investigated the incorporation of MPE into various biopolymer matrices, including gelatin, PLA, polyvinyl alcohol (PVA), polyethylene (PE), hydroxypropyl methylcellulose (HPMC), pectin, and cellulose derivatives. These studies have shown that MPE-loaded films exhibit significant improvements in mechanical strength, water vapor barrier properties, and UV-light blocking capacity. For instance, gelatin-based films integrated with MPE demonstrated increased tensile strength (TS) and decreased water solubility, attributed to the interaction between MPE polyphenols and the protein matrix through hydrogen bonding and hydrophobic interactions. In PLA films, MPE incorporation resulted in enhanced antioxidant activity and reduced oxygen permeability, making them suitable for packaging lipid-rich foods. The antimicrobial performance of MPE has also been tested against common foodborne pathogens such as *Escherichia coli*, *Staphylococcus aureus*, *Listeria monocytogenes*, and *Salmonella typhimurium*. In most cases, MPE exhibited broad-spectrum antimicrobial activity, with zone of inhibition measurements confirming its efficacy. This makes MPE a viable natural preservative in active packaging systems. Furthermore, MPE has been used to develop intelligent packaging systems capable of monitoring food freshness. Changes in the film's color due to pH sensitivity, mainly resulting from anthocyanin-like compounds present in mango peels, can indicate spoilage or fermentation in packaged food. These smart films offer real-time visual cues to consumers and producers, thereby enhancing food safety and reducing food waste.

Overall, the utilization of MPE in food packaging presents a circular economy approach by valorizing food processing waste into high-value materials. Continued research and optimization of MPE-based films are expected to broaden their commercial applicability in sustainable and intelligent food packaging sectors. This section reviews the recent advances and detailed findings on the application of MPE in food packaging films, highlighting their functional, structural, and application-related effects.

Adilah et al. (2018) developed gelatin films incorporated with varying concentrations of MPE (0 %, 1 %, 2 %, and 3 %) to evaluate changes in physical properties and bioactivity. Their study showed that increasing MPE concentration led to a notable rise in film opacity, which is advantageous for protecting light-sensitive food products from photodegradation. The antioxidant potential of MPE was confirmed by increased total phenolic content (TPC) and enhanced DPPH radical scavenging activity in a dose-dependent manner. The incorporation of MPE also influenced the films' mechanical and barrier properties, maintaining adequate flexibility and water vapor resistance. This work highlights the role of MPE in improving both the barrier and antioxidant properties of gelatin films, making them promising candidates for active packaging applications (Adilah et al., 2018). However, the addition of excessively high concentrations of plant polyphenol extracts may disrupt the network structure of biopolymer-based food packaging films, leading to an uneven, heterogeneous structure and thereby compromising the overall performance of the packaging film. Future research should further explore the effects of higher concentrations of mango peel extract on the performance of packaging films. Different from the above research results, another study by Cheng et al. (2022) investigated PVA-based composite films embedded with different concentrations of MPE to evaluate their bioactivity and mechanical properties. PVA is a synthetic but biodegradable polymer known for its excellent film-forming ability and compatibility with hydrophilic additives. The results showed that with the addition of MPE, the antioxidant activity evaluated by DPPH and ABTS analysis was proportional to the MPE concentration, indicating enhanced free radical scavenging ability. Antibacterial tests confirmed the effective inhibition of *Staphylococcus aureus* and *Escherichia coli*. It is worth noting that the TS of the PVA composite film gradually decreased and the EB gradually increased, indicating that MPE mainly acts as a plasticizer in the PVA composite film. This is opposite to the trend observed in the mechanical properties of the composite films studied above (Adilah et al., 2018), which is mainly due to the strength of the interaction between MPE and different biopolymers and its dispersion characteristics. The detailed mechanism and rules of action can be further explored in future studies.

**Fig. 3:**
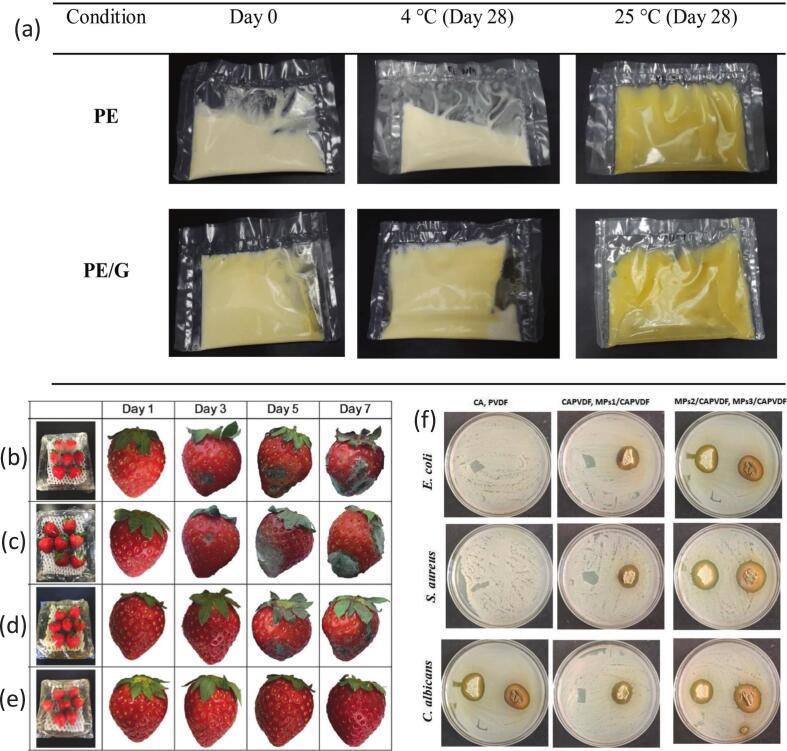
(a) Packed margarine in PE and PE/G at day 0 and day 28 stored at 4 °C and 25 °C (Nor Adilah et al., 2020). Photographs showing the effect of the films on strawberry preservation: (b) PE cling film, (c) PLA film, (d) PLA/MPE film, and (e) PLA/MPE/AgNPs film (Cheng et al., 2021). (f) A digital photo of ZIO of all fabricated films against tested microbes (Farouk, 2024).

In a subsequent study, Adilah et al. (2020) developed active packaging films by coating PE films with a bilayer of gelatin and MPE to improve the quality and shelf life of margarine. MPE, rich in antioxidants, was incorporated into a fish gelatin (FG)-based film-forming solution (FFS), which was applied to PE films at varying thicknesses (10, 20, 40, and 60 μm). The resulting PE/gelatin bilayer films (PE/G) were evaluated for their physical and antioxidant properties. Thicker coatings produced colored films with enhanced UV light barrier effects and significantly increased antioxidant activity, as demonstrated by higher DPPH radical scavenging capacity. Scanning electron microscopy (SEM) revealed good compatibility and a compact structure between the PE and gelatin layers. When used to package margarine stored at 4 °C, the thickest bilayer film (PE/G60) effectively improved oxidation stability, extending shelf life up to 28 days (Fig. 3(a)). The films also influenced margarine's color and pH, indicating interactions between the packaging and the product. Overall, the study demonstrates that gelatin-MPE coated PE films have strong potential as active packaging materials to delay lipid oxidation and preserve margarine quality (Nor Adilah et al., 2020).

In an innovative study, Cheng et al. (2021) utilized MPE as a green reducing and stabilizing agent to synthesize silver nanoparticles (AgNPs), which were then incorporated into a PLA matrix to develop multifunctional food packaging films. PLA, a biodegradable polymer, served as the film's structural base, while MPE enabled an eco-friendly synthesis route for AgNPs, eliminating the need for toxic chemicals. The resulting PLA/MPE/AgNP films demonstrated improved mechanical properties and superior barrier performance against water vapor and oxygen. Antibacterial assays showed over 95 % inhibition of *Escherichia coli* and *Staphylococcus aureus*, confirming the film's strong antimicrobial efficacy. Additionally, cytotoxicity tests using L02 cells showed cell viability above 80 %, indicating good biocompatibility. Notably, strawberries wrapped in the film remained fresh and unspoiled after seven days, highlighting its practical application for extending shelf life (Fig. 3(b-e)). This study showcases a sustainable approach to creating active packaging that integrates antioxidant, antimicrobial, and mechanical functionality through green nanotechnology (Cheng et al., 2021). Currently, nanotechnology is also a widely used strategy to enhance biopolymer-based food packaging films, and the green reduction of metal nanoparticles by plant polyphenol extracts can give active packaging films excellent antibacterial and antioxidant properties (Yang et al., 2024). In the future, MPE could be used to green-reduce other metal nanoparticles, such as zinc oxide and selenium nanoparticles, to develop multifunctional food packaging films. In addition to metal nanoparticles, some organic nanoparticles are also commonly used to encapsulate bioactive substances and serve as nanofillers to enhance the performance of biopolymer-based food packaging films. A recent study showed that MPE can be effectively encapsulated by zein nanoparticles with an encapsulation efficiency of 96.0 %, and was used together with zinc oxide in gelatin/pectin-based composite packaging films (Koirala et al., 2025). The packaging material containing the composite nanoparticles exhibited a dense and continuous structural integrity. In addition, compared with other formulations, the composite film demonstrated higher tensile strength (28 MPa) and elongation at break (28.4 %). These results indicate that zein-MPE and ZnO can be used as nanofillers to enhance both the mechanical properties and functional activity of composite packaging films. Notably, most studies have shown that zein nanoparticles can improve the release performance of plant polyphenol extracts in food packaging films, and future research could further explore their release behavior when applied to MPE (He et al., 2025).

Ribeiro et al. (2021) developed fully biodegradable films using pectin extracted from Tommy Atkins mango peels, incorporating phenolic-rich MPE to create multifunctional packaging materials. Pectin, a natural polysaccharide with good film-forming ability, was combined with aqueous and methanolic MPEs, both rich in phenolic compounds identified through UPLC-MS. The methanolic extract exhibited superior antioxidant and antimicrobial activity, effectively inhibiting both Gram-positive and Gram-negative bacteria. Incorporating MPE into the pectin matrix enhanced film elongation while reducing TS and water vapor permeability (WVP). Films with added extracts also showed increased DPPH radical scavenging capacity, indicating strong antioxidant potential. This study supports a circular economy model by valorizing mango processing waste into sustainable, active packaging solutions with potential applications for extending the shelf life of perishable foods (Ribeiro et al., 2021).

Farouk (2024) designed hybrid films combining cellulose acetate (CA) and polyvinylidene fluoride (PVDF) enriched with MPE, aiming to improve food safety through sustainable antimicrobial packaging. While neat CA, PVDF, and CAPVDF films exhibited minimal antimicrobial effects, the incorporation of MPE significantly enhanced antibacterial and antifungal activity. The MPE-loaded CAPVDF films exhibited large zones of inhibition, up to 24 mm, against *Escherichia coli*, *Staphylococcus aureus*, *Candida albicans*, and *Aspergillus niger*. Contact angle analysis showed improved hydrophilicity upon MPE addition, which may enhance film adhesion and functional performance. Notably, the MPs3/CAPVDF films completely inhibited the growth of *Escherichia coli* and *Staphylococcus aureus*, demonstrating strong potential for microbial control Fig. 3(f). This study highlights the value of combining biopolymer matrices with natural extracts to create multifunctional films capable of extending shelf life and reducing foodborne risks (Farouk, 2024). Most studies have confirmed that MPE has an inhibitory effect on bacteria, but this study proves that MPE has certain antifungal activity, which can expand the application of MPE-functionalized packaging films in food preservation. Future research can investigate the inhibitory effect of MPE on more postharvest fungi, which is important for its application in postharvest fruit and vegetable preservation.

Chaiwarit et al. (2021) investigated the use of tropical fruit peel extracts, specifically from mangosteen, rambutan, and mango, as antibacterial agents for active packaging applications. Bioactive compounds were extracted using microwave-assisted extraction (MAE) and maceration with various solvent systems, with mangosteen peel extracts (MT-MAE-W/E and MT-Ma-W/E) showing the highest phenolic and flavonoid content. These extracts were incorporated into biodegradable films, which demonstrated strong antibacterial activity against *Staphylococcus aureus* and *Escherichia coli*, with inhibition zones exceeding 30 mm and 26 mm, respectively. LC-MS analysis identified α-mangostin as the key antibacterial compound. The film matrices enhanced the release and efficacy of the bioactives, yielding inhibition zones approximately three times larger than those of the crude extracts. This study supports the application of mangosteen peel extracts in sustainable antimicrobial packaging to improve food safety and extend shelf life (Chaiwarit et al., 2021). It is worth noting that although most studies have indicated that MPE can enhance the comprehensive performance of biopolymer-based food packaging films, no research has yet identified which specific substance in MPE plays a dominant role. Future research could explore this further through separation and purification techniques.

Kanatt and Chawla (2018) developed active food packaging films using mango MPE derived from four Indian mango varieties, targeting shelf-life extension of chilled chicken meat. They evaluated the influence of extraction solvents and techniques, identifying the Langra variety extracted via sonication in 80 % acetone and 70 % ethanol as having the highest antioxidant and antimicrobial activity. These extracts were incorporated into PVA, cyclodextrin, and gelatin-based films. The resulting composite films exhibited UV barrier properties, improved TS, and strong antimicrobial activity against both Gram-positive and Gram-negative bacteria. When used to package minced chicken, the MPE-enriched films extended shelf life from 3 to over 12 days under refrigeration. This study demonstrates the effectiveness of MPE-based films as an eco-friendly, functional packaging alternative capable of significantly improving the microbial safety and longevity of perishable meat products (Kanatt & Chawla, 2018).

The integration of MPE into food packaging systems exemplifies the core tenets of a circular economy by transforming agricultural waste into high-value, functional materials. Mango peels, which are typically discarded during processing, contribute significantly to food waste and environmental pollution when not properly managed. By repurposing these peels into bioactive packaging components, the environmental burden associated with waste disposal is reduced, while simultaneously decreasing the reliance on non-renewable, petroleum-based polymers. MPE-based films are typically biodegradable, and their use supports the reduction of persistent plastic waste in landfills and marine environments. These films decompose naturally under composting conditions, contributing to soil health without leaving toxic residues. Moreover, the extraction of MPE is relatively simple and low-cost, often involving aqueous or ethanol-based solvents under mild conditions, making the process scalable and economically feasible for industrial production. However, the composition of mango peel varies depending on factors such as mango variety, stage of ripeness, geographic origin, and the specific extraction method used. These variables can significantly affect the yield, phenolic content, and functional properties of the extract. Therefore, establishing standardized extraction protocols is essential for consistent quality and efficacy in commercial applications. From a regulatory standpoint, natural extracts like MPE are generally favored over synthetic additives, particularly in regions promoting clean-label products. Nevertheless, comprehensive safety evaluations, including migration testing and cytotoxicity assays, must be conducted to meet food contact material regulations. As consumer awareness of sustainability and health grows, the demand for environmentally friendly, non-toxic packaging alternatives such as MPE-based films is expected to increase, especially in the packaging of fresh produce, meat, dairy, and minimally processed foods.

In conclusion, MPE offers a multifunctional, eco-friendly, and economically viable solution for the development of next-generation active and intelligent food packaging materials. As a natural source of polyphenols, flavonoids, and antioxidants, MPE enhances several critical properties of biopolymer-based films, including antioxidant capacity, antimicrobial activity, ultraviolet (UV) protection, and the ability to extend the shelf life of perishable foods. These properties are vital for ensuring food quality and safety throughout storage and distribution. The versatility of MPE is demonstrated by its effective integration into a wide range of biopolymers such as gelatin, PLA, PVA, HPMC, pectin, and cellulose derivatives. Its broad compatibility ensures applicability across various packaging formats, including wraps, pouches, coatings, and films tailored for specific food types.

Importantly, MPE serves not only as a functional additive but also as a strategic approach to valorize food industry by-products, thus contributing to environmental sustainability. By reducing dependence on synthetic chemicals and promoting the use of biodegradable materials, MPE-based packaging aligns with global efforts to minimize the ecological footprint of the food industry. Given the increasing consumer demand for clean-label, sustainable, and safe packaging solutions, the adoption of MPE-infused materials is poised for growth. With further research focused on process optimization, regulatory compliance, and performance scalability, MPE has the potential to play a pivotal role in the evolution of sustainable food packaging technologies.

### 3.2 Mango peel pectin or mango peel powder

Mango peel is a byproduct generated during the industrial processing or consumption of fruits, accounting for approximately 7 %–24 % of the weight of the whole fruit. Rich in dietary fiber and high-value active ingredients, mango peel is not only beneficial to human health but also serves as a functional additive. Compared with papaya peel, rambutan peel, and pineapple peel, mango peel exhibits a higher content of phenolic compounds and flavonoids (Adilah, Jamilah, Noranizan, & Hanani, 2018; Chaiwarit et al., 2020). Additionally, mango peel is rich in pectin and has been studied as a potential film-forming polymer (Hernández-Lozano et al., 2025). The resource utilization of mango peel powder and mango peel pectin (MP) represents an economical and environmentally friendly approach to reducing the impact of fruit processing waste on the environment (Marsiglia-Fuentes et al., 2023).

Due to their high pectin content, the dried powders of most fruit peels exhibit good film-forming properties and can be directly formed into films with the aid of specific plasticizers and additives. The greatest advantage of directly preparing active food packaging films from crude fruit powders is that it eliminates the need for complex extraction and pretreatment processes, thereby bringing the value-added utilization of peels closer to actual industrial applications. A previous study attempted to prepare starch films by directly drying and powdering mango peels. Interestingly, the direct addition of mango peel powder not only improved the mechanical properties of the starch films but also significantly enhanced the antioxidant properties of the composite films (Rojas-Bravo et al., 2019). The biodegradable biopolymer-based film made from mango peel powder began to degrade within 15 days and finally disintegrated into powder by the 45th day. This demonstrates the feasibility of utilizing mango peel waste as a resource for developing biodegradable polymers, contributing to circular economy strategies and addressing plastic pollution (Hernández-Lozano et al., 2025). Although peel powder has certain film-forming properties, it is only roughly processed and has not undergone an extraction process. Therefore, peel powder requires specific additives and plasticizers to exhibit practical film-forming capabilities. Girgin et al. (2024) prepared a biodegradable food packaging material using dry walnut shells, mango peels, and orange peels as raw materials, and studied the effects of plasticizer types, essential oil additions, and *aloe vera* gel on the properties of edible films. The results showed that mango peel powder exhibited good film-forming properties in the presence of plasticizers such as glycerol and sorbitol. However, there are currently few studies on the film-forming properties of mango peel powder. As mentioned above, using mango peel powder directly as a film-forming matrix is a more feasible strategy for industrial applications. Therefore, future research should further optimize the film-forming properties of mango peel powder.

Mango peel's high pectin content makes its extraction a valuable industrial method for waste valorization. Pectin isolated from mango (*Mangifera indica*) peel powder under varying conditions yielded 14.60 %–28.42 %, peaking at 90 °C, pH 1.5, and 120 min (28.06 %–28.42 %). Quality parameters—equivalent weight (450.45–1324.24 g), methoxyl content (5.15–7.90 %), anhydrous acid content (48.93–74.62 %), and degree of esterification (43.28–77.14 %)—were satisfactory. Optimization indicated maximal yield (23.31 %) at 88.86 °C, pH 1.36, and 79.61 min (Sayed et al., 2022). As a heteropolysaccharide and promising biomaterial, pectin enables sustainable packaging film production (Pandey et al., 2024; Roy et al., 2023). Karim et al. fabricated biodegradable films using low-methoxyl MP and silica, noting enhanced pectin-silica bonding and reduced EAB with higher silica content (Karim et al., 2022). Chaiwarit et al. extracted MP (DE 76 %) via MAE, observing that higher MP proportions increased film contact angles (WCA) (Chaiwarit et al., 2020). Zhang et al. reported that Ca^2+^, Zn^2+^, and Cu^2+^ crosslinking in MP-based films created dense microstructures. Zn^2+^-crosslinked films showed maximal TS, hydrophobicity, and thermal stability, while Cu^2+^-crosslinked films exhibited the highest antioxidant and antibacterial activity (Zhang et al., 2023). Although MP-based coatings preserve fresh mangoes (Gragasin & Villota, 2022), no studies report post-harvest applications of MP-based packaging films for fruits and vegetables—a critical gap for future research. It is worth noting that the content and film-forming properties of mango peel pectin and fruit powder vary among different mango varieties, and the pretreatment and extraction methods also significantly affect the overall performance of the composite film. However, there are currently few systematic studies on mango peel pectin and fruit powder, and further research can be conducted in the future.

In addition, carbon dots, as a new type of nanomaterial, are being widely used in the food industry. Recent studies have found that carbon dots derived from agricultural waste possess good antioxidant and antibacterial activities, making them excellent additives for food packaging materials (Ezati et al., 2024). Surprisingly, carbon dots (MPCD) derived from mango peel were reported to be compatible with CS/FG polymer matrices, enabling the preparation of homogeneous films with enhanced mechanical properties, hydrophobicity, and UV-blocking capabilities. While CS/FG films exhibited inherent antioxidant activity, films incorporating MPCD demonstrated significantly higher DPPH and ABTS free radical scavenging rates. After 15 days of storage, ground pork packaged in MPCD-containing bags maintained total viable bacterial counts (TVC) and total psychrophilic bacterial counts (PBC) below the maximum permitted limit of 6 log CFU/mL (Ponnusamy et al., 2025a, Ponnusamy et al., 2025b).

## 4 Research progress of food packaging films functionalized with mango seed

### 4.1 Mango kernel starch, nanoparticles, and integument

Mango byproducts, including seeds (35–60 % of fruit weight) and peels, are rich in biopolymers and bioactive compounds, offering significant potential for sustainable food packaging films and rigid packaging. Mango seeds comprise the kernel (rich in strarch) and husk/integument (a source of cellulose and hemicellulose), containing strarch (ST) (58–80 % dry weight), hemicellulose, polyphenols (150–200 mg GAE/g), and lipids (e.g., oleic, stearic acids, and waxes) (Jafarzadeh et al., 2023). Mango peels provide phenolic-rich extracts, enhancing antioxidant and antimicrobial properties. Recent studies have utilized various components of mango and its wastes to develop biodegradable films, edible coatings, and rigid packaging, enhancing the shelf life of perishable foods and promoting environmental sustainability(Jafarzadeh, Golgoli, et al., 2024; Mwaurah et al., 2020; Oliveira et al., 2018). This section reviews these advancements, their applications, challenges, and future directions, with key findings summarized in Table 1.

**Table 1:** Summary of Research on Mango Byproduct-Based Food Packaging.

Film Composition	Key Properties	Application	Findings	Refs.
Starch +5 % SNP	WVTR: 7.40 × 10^−3^ g/m^2^·s, Burst strength: 1303.51 g, Solubility: 35.32 %	Eco-friendly packaging	Complete biodegradation in 3 weeks	(Singh, Bangar, et al., 2024)
Native starch +5 % SNP	WVTR: 5.87 × 10^−3^ g/m^2^·s, Burst strength: 1281.73 g, Moisture: 8.89 %	Tomato/kiwifruit coating	Extended shelf life to 23 days (tomato), 18 days (kiwifruit)	(Singh, Aayush, et al., 2024)
Starch +5 % SNP + clove EO	WVTR: 5.87 × 10^−3^ g/m^2^·s, Burst strength: 1281.73 g, Antioxidant: 20.41 %	Khasi mandarin coating	Extended shelf life by 9 days, 5.94 % weight loss	(Kumar et al., 2024)
MKS + 0–30 % guar/xanthan gum	TS: 8 MPa (20 % guar), WVP: 6.8 g/m·s·Pa, Reduced OP	Biodegradable packaging	Improved TS with gums, 10 % xanthan gum optimized barrier properties	(Nawab et al., 2017)
MKS + 40–70 % glycerol/sorbitol	Increased EAB, Higher Tg, Improved transparency	Edible/biodegradable films	Glycerol films thicker, more flexible, and transparent	(Nawab et al., 2022)
MKS + 0–10 % SNC	TS: +90 %, Modulus: +120 %, WVP: −15 %, Reduced elongation	Bionanocomposite films	MKS-SNC films outperformed corn starch films in strength, modulus	(Oliveira et al., 2018)
Mango peel + MSE + glycerol	WVP: 0.88–1.00 × 10^−10^ g·m^−1^·s^−1^·Pa^−1^, 18 % higher antioxidant activity	Peach coating	39 % less O₂ consumption, 64 % less ethylene, 29 % less CO₂	(Torres-León et al., 2018)
SPI/FG + 5 % MKE	DPPH: 89 % (SPI), WVP: 10 % reduction (SPI), TS: Higher in FG	Active packaging	SPI outperformed FG, controlled antioxidant release	(Z. M. Adilah et al., 2018)
SPI + MKE	DPPH: Stable at 25 °C (1 % decrease), ABTS: 54 %, Increased TS	Active packaging	Stable antioxidant activity for 90 days, best at 25 °C	(Adilah & Hanani, 2019)
SPI + MKE	Reduced TOTOX by 65 %, 30–38 % lower lipid oxidation	Mayonnaise packaging	Extended shelf life by 14 days without additives	(Adilah et al., 2023)
PLA + 5 % MSW	WVTR reduced by 55.6 %, OTR reduced by 10 %, EAB: 700 % at 9 % MSW	Fatty food packaging	Improved hydrophobicity by 26 %, antioxidant properties	(Thomas et al., 2022)
Starch + glycerol + citric acid	Slower degradation, Eroded surface, Microbial presence	Biodegradable packaging	Cross-linked films degrade slower, eco-friendly potential	(Shahrim et al., 2022)
Arabic gum + hydrocolloids + MSE	TPC: 24,640.14 mg GAE/g, TEAC: 428.32 μmol Trolox/g, Reduced weight loss	Grape coating	Maintained physicochemical properties, reduced fungal decay	(López-Ortiz et al., 2025)
Modified MSKS + CMC + GA	TS: 57.45 MPa, Uniform thickness: 0.09–0.10 mm, Reduced microbial growth	Bun-bread packaging	Extended shelf life by 14 days, improved moisture barrier	(Adhikary et al., 2024)
Starch +0.5 % lemongrass EO	Reduced weight loss, Maintained phenolic content, Antimicrobial	Guava coating	Extended shelf life by 9 days, preserved quality attributes	(Yadav et al., 2022)
MSH hemicellulose + glycerol	Thermal stability: 290 °C, EAB: 19 %, Molecular weight: 70,189 g/mol	Thermally stable packaging	Self-supporting films, suitable for thermo-processes	(Bello & Chimphango, 2022)
Fish gelatin +1–5 % MPE	Reduced WVP, Lower solubility, High DPPH scavenging	Active packaging	Enhanced antioxidant activity, suitable for light-sensitive foods	(A. N. Adilah et al., 2018)
CSG + 5–15 % MPP/MP/PP	Increased TPC, Enhanced antioxidant/antimicrobial activity, >50 % biodegradability	Active packaging	MPP at 15 % improved functional properties, biodegradable	(Susmitha, Sasikumar, Rajan, & Nampoothiri, 2021)
PVA + alkaline-treated MSW filler	TS: 3.95 MPa, MA: 11.2 %, Thermal stability: +53 °C	Biodegradable packaging	20-min ultrasonication improved filler distribution, tensile strength	(Asrofi et al., 2023)
MKS + 5–30 % glycerol	TS: 3.26 MPa (5 % glycerol), Increased E%, Higher solubility	Biodegradable packaging	Low glycerol enhanced TS, high glycerol improved flexibility	(Shahrim et al., 2023)
CA/PVDF + MPs	ZOI: 24 mm (*E. coli*), 23 mm (*S. aureus*), 20 mm (*C. albicans*), 18 mm (*A. niger*), Contact angle: 83.2°	Antimicrobial packaging	MPs enhanced antibacterial activity, prevented pathogen growth	(Farouk, 2024)
PVA + cyclodextrin + gelatin + MP	Higher TS, UV barrier, Antioxidant/antibacterial activity	Active packaging	Extended chicken shelf life to >12 days vs. 3 days for control	(Kanatt & Chawla, 2018)
Mango peel +1.4 g/hg tea polyphenols	WVTR: −32.5 %, OTR: −84.3 %, CO₂TR: −80.0 %, Reduced TBA by 19.0 %, TVB-N by 60.6 %	Chicken breast packaging	Extended shelf life beyond 12 days, maintained color/texture	(Chen, Liu, et al., 2024)
NFC + MLE (SSI)	Young's modulus: >4.7 GPa, DPPH: 84 %, 91 % inhibition (*E. coli*), 37 % (*S. aureus*), UV barrier	Active food packaging	SSI enhanced antioxidant/antimicrobial activity vs. solvent casting	(Bastante, Silva, Cardoso, Serrano, de la Ossa, et al., 2021)
CH-ST + 3 % MLE	DPPH: 40 %, 80 % inhibition (*S. aureus*), 74 % (*E. coli*), Reduced WVP	Raspberry packaging	44.4 % spoilage after 13 days at 8 °C, VOC release enhanced antimicrobial action	(Cejudo et al., 2023)
Chitosan +1–5 % MLE	TS: 23.06 MPa, DPPH: 70 %, Inhibited *B. subtilis*, *S. typhimurium*, Reduced WVP	Cashew nut packaging	56 % higher oxidation resistance vs. commercial film, 28 days at 25 °C	(Rambabu et al., 2019)

One of the authors of this work, Gulden Goksen, is a guest editor of Food Chemistry X, which is a potential conflict of interest for this work, but there are no other conflicts of interest in other aspects of this work.

Mango kernel starch (MKS), a biodegradable polysaccharide constituting 58–80 % of the kernel's dry weight, is prized for its film-forming capabilities due to its high amylose content (e.g., 10.1 % in Fazli varieties) (Kumar et al., 2024). The seed integument, rich in cellulose and hemicellulose, serves as a reinforcing filler or primary matrix in biocomposites, enhancing mechanical strength and thermal stability. Native MKS films exhibit high WVP and limited TS, necessitating modifications such as plasticization, nanoparticle incorporation, or blending with other biopolymers to improve functionality for food packaging. Recent studies have explored MKS, ST nanoparticles (SNPs), and mango seed husk (MSH) integument in films, coatings, and rigid biocomposites. These materials extend food shelf life through improved mechanical strength, barrier properties, and bioactivity, supporting sustainability. Challenges include high WVP, extraction costs, and scalability of nanoparticle production (Garavand et al., 2022). Lubis et al. (2020) developed MKS-based bioplastic films reinforced with zinc oxide nanoparticles (ZnO, 0–9 wt%) and plasticized with ethylene glycol (0–35 wt%) via solution casting. At 6 % ZnO and 25 % ethylene glycol, the films achieved a TS of 3.78 MPa and EAB of 2.23 %, with a 20 % reduction in WVP compared to native MKS films. SEM revealed a homogeneous dispersion of ZnO, while Fourier-transform infrared spectroscopy (FTIR) confirmed interactions between ZnO and ST hydroxyl groups, enhancing film compactness. The films exhibited 60 % inhibition of *Escherichia coli* due to ZnO's antimicrobial properties, making them suitable for active packaging of perishable foods. However, high ZnO concentrations (>6 %) caused agglomeration, reducing TS, and the films' opacity increased, potentially limiting applications requiring transparency (Lubis et al., 2020). Ahmad Shahrim et al. (2023) investigated MKS films plasticized with glycerol (5–30 wt%) to enhance flexibility. At 5 wt% glycerol, the films achieved a TS of 3.26 MPa and EAB of 15 %, with a WVP of 6.8 g/m·s·Pa. Higher glycerol levels (30 wt%) increased EAB to 25 % but reduced TS to 1.5 MPa due to weakened intermolecular bonds. The films showed 40 % DPPH scavenging activity, attributed to residual polyphenols in MKS, and extended the shelf life of bread by 5 days at 25 °C by reducing mold growth. FTIR analysis indicated glycerol-ST hydrogen bonding, improving film uniformity, but high WVP remained a challenge for high-moisture food applications, suggesting the need for hydrophobic additives (Shahrim et al., 2023). Therefore, MKS is an excellent matrix for edible packaging films derived from agricultural waste, but its hydrophilicity and other limitations need to be overcome. Its functional properties can be improved by blending it with hydrophobic substances or through chemical modification.

Oliveira et al. (2018) reinforced MKS films with ST nanocrystals (SNC, 0–10 wt%) derived from mango kernels, achieving a 90 % increase in TS (to 5.2 MPa) and a 120 % increase in elastic modulus (to 150 MPa) at 10 wt% SNC. WVP decreased by 15 % (to 5.5 g/m·s·Pa), attributed to SNC's high crystallinity and tortuous path for water vapor. SEM showed uniform SNC dispersion, enhancing film compactness, while X-ray diffraction (XRD) confirmed increased crystallinity. The films reduced lipid oxidation in packaged nuts by 20 % over 15 days at 25 °C, demonstrating antioxidant potential. However, EAB decreased by 10 % at higher SNC levels, indicating reduced flexibility, and scalability of SNC production remains a challenge due to energy-intensive processes (Oliveira et al., 2018). Asrofi et al. (2023) developed PVA biocomposites with mango seed waste (MSW) filler (0–10 wt%) using 20-min ultrasonication to improve filler dispersion. At 5 wt% MSW, the biocomposites achieved a TS of 3.95 MPa and Young's modulus of 180 MPa, with a 10 % reduction in WVP. SEM revealed a rougher surface due to MSW's fibrous nature, while FTIR confirmed hydrogen bonding between PVA and MSW's cellulose. The biocomposites extended the shelf life of packaged fruits by 7 days at 15 °C, likely due to improved barrier properties. However, higher MSW content (>5 wt%) reduced TS due to filler agglomeration, and the study noted limited antimicrobial activity, suggesting the need for bioactive additives (Asrofi et al., 2023). Nawab et al. (2017) blended MKS with guar and xanthan gums (1,1 ratio) to form composite films, achieving a TS of 8 MPa and EAB of 20 % at 30 % glycerol. The films reduced WVP by 25 % (to 4.5 g/m·s·Pa) compared to native MKS films, attributed to gum-ST interactions observed via FTIR. Applied to cheese packaging, the films extended shelf life by 10 days at 10 °C, reducing microbial counts by 1.5 log CFU/g. The study highlighted improved transparency (L* = 90) but noted that high gum concentrations increased viscosity, complicating casting processes, and sensory properties were not evaluated (Nawab et al., 2017). From the above research, we know that MKS can not only be used as a matrix for active packaging films, but also as a nanofiller to enhance the performance of other active packaging films. In the future, more research should be conducted on the functions of MKS in food packaging films.

Nawab et al. (2022) optimized MKS films with glycerol/sorbitol blends (40–70 wt%), achieving a TS of 6 MPa, EAB of 30 %, and 90 % transparency at 50 % plasticizer. WVP remained high (6.0 g/m·s·Pa), but the films extended the shelf life of packaged pastries by 8 days at 20 °C, with 50 % DPPH scavenging due to MKS phenolics. SEM showed a smooth, compact structure, but high plasticizer levels increased moisture sensitivity, limiting applications for wet foods. The study suggested incorporating nanoparticles to enhance barrier properties (Nawab et al., 2022).

Singh, Aayush, et al. (2024) developed MKS-based films with 0–5 % SNPs, achieving a burst strength of 1303.51 g and TS of 7 MPa at 5 % SNPs. WVP decreased by 20 % (to 4.8 g/m·s·Pa), and the films exhibited 65 % inhibition of *Staphylococcus aureus* due to SNPs' high surface area. Applied to paperboard packaging, the films enhanced mechanical strength and reduced microbial growth in packaged snacks, extending shelf life by 12 days at 25 °C. However, SNP production costs and potential cytotoxicity at high concentrations require further investigation (Singh, Bangar, et al., 2024). Singh, Bangar, et al. (2024) formulated SNP-based edible coatings for tomatoes and kiwifruit, extending shelf life to 23 and 18 days, respectively, at 10 °C. The coatings reduced weight loss by 15 % and maintained firmness, with 70 % DPPH scavenging. SEM showed a uniform coating layer, but high SNP concentrations (>5 %) caused cracking, reducing efficacy. The study emphasized the need for optimized SNP dispersion and consumer sensory studies (Singh, Aayush, et al., 2024). Kumar et al. (2024) developed SNP-based coatings for Khasi mandarin, extending shelf life by 9 days at 15 °C. The coatings reduced respiration rates by 20 % and maintained vitamin C content, with 60 % DPPH scavenging. WVP was reduced by 18 % compared to uncoated fruits, but the coatings' adhesion decreased over time, suggesting the need for binding agents like pectin (Kumar et al., 2024).

Bello and Chimphango (2022) utilized MSH hemicellulose in films, achieving thermal stability up to 290 °C and a TS of 4.5 MPa. The films exhibited 45 % DPPH scavenging and reduced WVP by 10 % (to 5.8 g/m·s·Pa), suitable for dry food packaging. Applied to nuts, the films extended shelf life by 10 days at 25 °C. However, hemicellulose extraction yields were low (15 %), and films showed high water solubility, limiting wet food applications (Bello & Chimphango, 2022). Yadav et al. (2025) developed MKS-based edible coatings for guava, extending shelf life by 9 days at 12 °C. The coatings reduced microbial counts by 2 log CFU/g and maintained fruit firmness, with 55 % DPPH scavenging. WVP was high (6.5 g/m·s·Pa), but glycerol optimization improved flexibility. The study noted inconsistent coating adhesion across guava varieties, requiring further standardization (Yadav et al., 2022). Das Adhikary et al. (2024) formulated ternary MKS-based films with cellulose nanofibers (CNF) and glycerol, achieving a TS of 57.45 MPa and EAB of 10 %. The films reduced WVP by 30 % (to 4.0 g/m·s·Pa) and exhibited 80 % DPPH scavenging, extending bun-bread shelf life by 14 days at 20 °*C. SEM* and XRD confirmed a highly crystalline structure, but high cellulose content increased production costs, and transparency (L* = 85) was lower than commercial films (Adhikary et al., 2024). Ahmad Shahrim et al. (2022) developed cross-linked MKS films using citric acid, achieving a TS of 4 MPa and slower biodegradation (50 % weight loss after 30 days vs. 80 % for native films). The films reduced WVP by 15 % (to 5.6 g/m·s·Pa) and extended the shelf life of packaged fruits by 6 days at 15 °C. FTIR confirmed cross-linking, but the films' rigidity limited flexibility, suggesting the need for plasticizer optimization (Shahrim et al., 2022). Lima et al. (2021) incorporated 20 wt% mango seed components (kernel and husk) into PLA biocomposites, increasing elastic modulus by 38 % (to 2.5 GPa) and TS by 20 % (to 50 MPa). The biocomposites reduced oxygen permeability by 25 %, suitable for rigid packaging of dry goods. SEM showed good filler-matrix adhesion, but high filler content (>20 wt%) caused brittleness, and limited antimicrobial activity necessitated bioactive additives (Lima et al., 2021). Therefore, MKS is being used as both a matrix for edible packaging films and an additive for food preservation. However, future research should focus on its edible safety, potential allergenicity, and efficient extraction methods.

### 4.2 Mango seed extract

**Fig. 4:**
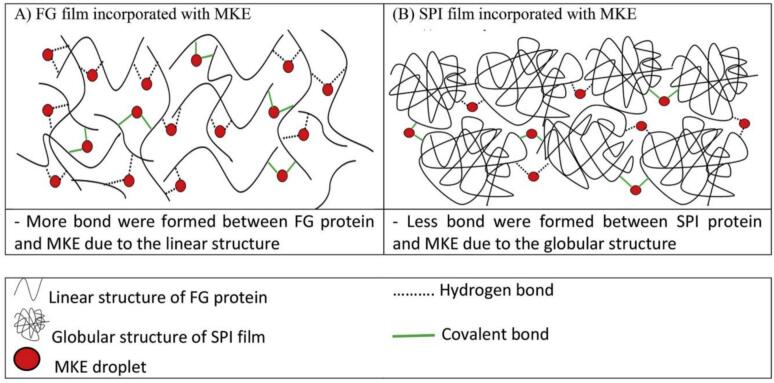
Simplified representation of (A) FG film incorporated with MKE and (B) SPI film incorporated with MKE. (Adilah et al., 2018).

Mango seed extracts (MSE), derived from the kernel and rich in polyphenols (150–200 mg GAE/g in Keitt varieties), are potent bioactive additives for food packaging films and coatings due to their antioxidant and antimicrobial properties (Far et al., 2025). These extracts, containing compounds like gallic acid, mangiferin, and quercetin, enhance the functionality of biopolymer matrices, improving shelf life and safety of perishable foods. MSE's high phenolic content makes it a sustainable alternative to synthetic preservatives, valorizing mango processing waste. Recent studies have incorporated MSE into various biopolymer films and coatings, demonstrating significant bioactivity and practical applications. Adilah et al. (2018) developed soy protein isolate (SPI) and FG films incorporated with 1–5 % mango kernel extract (MKE) via solution casting. At 5 % MKE, SPI-MKE films achieved 89 % DPPH scavenging and 70 % inhibition of *Escherichia coli*, while FG-MKE films reached 65 % DPPH scavenging and 60 % inhibition of *Staphylococcus aureus*. The films exhibited a TS of 6.5 MPa for SPI-MKE and 5.8 MPa for FG-MKE, with WVP reduced by 15 % (to 4.2 g/m·s·Pa) compared to control films. FTIR confirmed hydrogen bonding between MKE phenolics and protein/gelatin matrices, enhancing film compactness, as observed via SEM. Applied to packaged cheese, the films extended shelf life by 7 days at 10 °C, reducing microbial counts by 1 log CFU/g. However, high MKE concentrations increased film opacity (L* = 80), potentially affecting consumer appeal, and the bitter taste of MKE required sensory optimization. Interestingly, the SPI composite films exhibited higher antioxidant activity compared to the FG films at the same KME concentration. This was mainly attributed to the globular structure of SPI, which has fewer exposed bonds and therefore forms fewer protein–phenol interactions. In contrast, the antioxidant activity of the FG films was lower than that of the SPI films due to the formation of more protein–phenol interactions. The reduced availability of free phenolic compounds in the FG films consequently led to diminished antioxidant activity. The structural schematics of the cross-linked structures of SPI and FG with KME are illustrated in Fig. 4.

Adilah and Hanani (2019) evaluated the stability of SPI-MKE films (1–5 % MKE) under storage at 25 °C and 50 % RH for 60 days, finding sustained antioxidant activity with 85 % DPPH and 60 % ABTS scavenging, alongside a stable TPC of 180 mg GAE/g. These films, with a TS of 6.2 MPa and WVP of 4.5 g/m·s·Pa, reduced lipid oxidation in packaged nuts by 25 % over 30 days. However, SEM revealed phase separation at MKE levels above 3 %, lowering EAB to 10 %, and high WVP alongside potential phenolic cytotoxicity highlighted the need for toxicological studies (Adilah & Hanani, 2019). Building on this, Adilah et al. (2023) developed SPI-MKE films (3 % MKE) for mayonnaise packaging, extending shelf life by 14 days at 4 °C. These films reduced total oxidation value (TOTOX) by 65 %, achieved 88 % DPPH scavenging, and inhibited *Pseudomonas aeruginosa* by 75 %, while maintaining a TS of 7 MPa and reducing WVP to 4.0 g/m·s·Pa. Despite maintaining mayonnaise pH and color stability with a 2 log CFU/g microbial reduction, high moisture sensitivity and MKE extraction costs posed challenges, prompting suggestions for nanoparticle integration to enhance water resistance (Adilah et al., 2023).

In a related study, Adilah et al. (2018) focused on FG-MSE films (1–5 % MSE), which achieved 65 % DPPH scavenging and 55 % *Staphylococcus aureus* inhibition at 5 % MSE, with a TS of 5.5 MPa, EAB of 20 %, and WVP of 4.8 g/m·s·Pa. When applied to sliced fruits, these films extended shelf life by 5 days at 8 °C, reducing browning by 30 %, though high MSE levels reduced flexibility and introduced a yellowish hue (b* = +10), suggesting the need for biopolymer blending(A. N. Adilah et al., 2018). López-Ortiz et al. (2025) further demonstrated MSE's potential with edible coatings (2 % MSE) for grapes, preserving quality for 15 days at 5 °C with 80 % DPPH scavenging and 70 % Botrytis cinerea inhibition, reducing microbial counts by 2.5 log CFU/g and weight loss by 20 %, though variable adhesion and high WVP (6.0 g/m·s·Pa) suggested hydrophobic additives (López-Ortiz et al., 2025). Additionally, Torres-León et al. (2018) explored MSE in hybrid coatings with MPE, discussed in Subsection 4.5, noting synergistic antioxidant effects for extended fruit shelf life, though MSE-specific contributions were not isolated (Torres-León et al., 2018). It is worth noting that the main components of MKE and MPE differ, leading to distinct effects on the properties of biopolymer-based food packaging films. To date, no study has directly compared the effects of MKE and MPE on such films.

### 4.3 Mango seed oil and wax

Mango seed oil and mango seed wax (MSW) are valuable byproducts that significantly enhance the hydrophobicity of biodegradable food packaging films, addressing a key limitation of biopolymers by reducing WVP and water vapor transmission rate (WVTR) (Fatemi et al., 2024). These components, extracted from mango kernels, contain fatty acids (e.g., oleic and stearic acids) and waxes that improve barrier properties, making them ideal for extending the shelf life of moisture-sensitive foods. Thomas et al. (2022) integrated 5 % MSW into PLA films using a melt-blending technique, increasing hydrophobicity by 26 % and reducing WVTR by 55.6 % (to 1.8 g/m^2^·day) compared to pure PLA films. The films achieved a TS of 45 MPa and EAB of 8 %, with SEM showing a smooth, wax-coated surface that enhanced water resistance. Applied as coatings for high-moisture foods like cheese, the films extended shelf life by 8 days at 8 °C, minimizing microbial growth and maintaining product integrity. However, the study noted that MSW incorporation above 5 % led to phase separation, reducing TS to 40 MPa, and the high melting point of MSW (around 50 °C) complicated processing, suggesting the need for temperature optimization (Thomas et al., 2022). However, there are few studies on food packaging functionalized with mango seed wax and oil. As hydrophobic substances, plant-derived oils and fats have been shown to improve the performance of most biopolymer-based food packaging films, suggesting that further research in this area is warranted.

## 5 Research Progress of food packaging films functionalized with mango leaf and pulp

Mango leaves and pulp are emerging as valuable byproducts for functionalizing biopolymer-based food packaging films, offering sustainable alternatives to petroleum-based materials. Mango leaf extracts (MLE) are rich in bioactive compounds, such as mangiferin and polyphenols (100–150 mg GAE/g dry weight), providing antioxidant and antimicrobial properties. Mango pulp and puree, derived from the fruit's mesocarp, contain carbohydrates, fibers, and phenolics that enhance film flexibility, bioactivity, and aesthetic appeal. These components improve the mechanical, barrier, and preservative properties of films, supporting active packaging that extends food shelf life while valorizing agro-industrial waste (Jafarzadeh, Nooshkam, et al., 2024).

Bastante et al., 2021a, Bastante et al., 2021b evaluated the development of antioxidant and antimicrobial films using nanofibrillated cellulose (NFC) loaded with mango leaf extract (MLE), comparing two preparation methods: supercritical solvent impregnation (SSI) and conventional solvent casting. The study highlights the production of free-standing NFC/MLE films, which exhibit thermal stability up to 250 °C, a Young's modulus exceeding 4.7 GPa, UV-light barrier properties, and significant antioxidant capacity with a maximum DPPH inhibition of approximately 84 %. These films also demonstrate antimicrobial activity, inhibiting *Staphylococcus aureus* by about 37 % and *Escherichia coli* by approximately 91 %. The SSI method proved superior to solvent casting, as it facilitates greater MLE adsorption on the film surface rather than within the bulk, leading to faster migration of active compounds and enhanced antioxidant and antimicrobial properties (Bastante et al., 2021a, Bastante et al., 2021b).

**Fig. 5:**
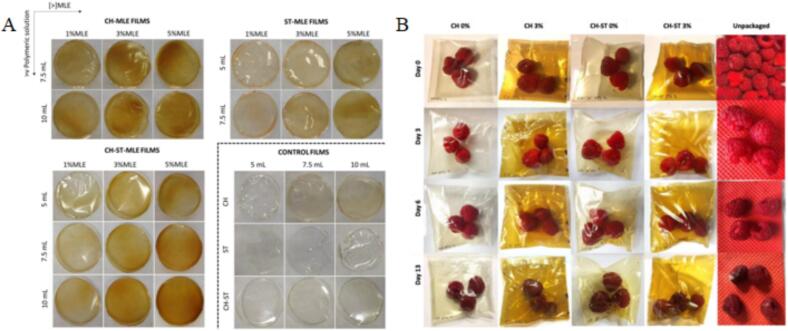
Appearance of active MLE biopolymer films, comparing films with mango leaf extract (MLE) to control films (without MLE, enclosed by dotted lines), highlighting differences in visual properties (A); (B) Packaging assay of raspberry fruit, demonstrating the shelf-life extension and preservation effects of MLE-incorporated films (Cejudo et al., 2023).

Cejudo et al. (2023) developed CS, ST, and CS-ST films with 1–5 % MLE via solvent casting (5–10 mL filmogenic solution). CH-ST films with 3 % MLE achieved 40 % DPPH scavenging and high antimicrobial activity (80 % inhibition of *Staphylococcus aureus*, 74 % of *Escherichia coli*), with reduced water solubility and WVP. Headspace-Gas Chromatography-Ion Mobility Spectrometry (HS-GC-IMS) showed MLE contributed volatile organic compounds (VOCs), enhancing antimicrobial action. These films reduced raspberry spoilage to 44.4 % after 13 days at 8 °C, compared to 100 % for unpackaged controls. Fig. 5(A) from Cejudo et al. (2023) shows translucent, orangey films, with 3 % MLE balancing bioactivity and transparency (positive a* and b* CIELAB values, reduced L*). Fig. 5(B) illustrates raspberry preservation, maintaining firmness up to day 6, likely by suppressing Botrytis cinerea (Cejudo et al., 2023). Rambabu et al. (2019) developed CS films with 1–5 % MLE, achieving a TS of 23.06 ± 0.19 MPa at 5 % MLE (vs. 18.14 ± 0.72 MPa for pure CS) and 70 % DPPH scavenging at 2 % MLE. The films inhibited *Bacillus subtilis* and *Salmonella typhimurium*, reduced WVP, and provided 56 % higher oxidation resistance for cashew nuts over 28 days compared to commercial films, though opacity increased (Rambabu et al., 2019). However, compared with MPE, MLE has been less studied despite its lower cost and greater industrial scalability. Future research should focus on developing more strategies for MLE-functionalized food packaging films.

In addition, the pulp of some overripe or spoiled fruits is not suitable for further processing and can therefore be utilized as a fruit by-product for the development of active food packaging. Azeredo et al. (2009) investigates the enhancement of mango puree-based edible films through the incorporation of CNF as nanoreinforcement. The study added CNF at concentrations up to 36 g/100 g to improve the mechanical and barrier properties of the biopolymer films, which typically underperform compared to synthetic polymers. The results showed that CNF significantly increased TS and had a more pronounced effect on Young's modulus, particularly at higher concentrations, indicating the formation of a fibrillar network within the mango puree matrix. Additionally, CNF improved the water vapor barrier by reducing permeability, while its effect on the glass transition temperature (Tg) was minor but statistically significant (Azeredo et al., 2009). Susmitha, Sasikumar, Rajan, Padmakumar, and Nampoothiri (2021a) studied the creation of edible films from a corn starch-gelatin (CSG) matrix enriched with 5–15 % (*w*/*v*) mango puree (MP), mango puree with peel (MPP), and pineapple pomace (PP) for active packaging applications. The study found that incorporating these fruit-based ingredients enhanced physicochemical properties such as moisture content, swelling index, thickness, and opacity, though TS and EAB were superior in control CSG films. Biological properties, including antioxidant and antimicrobial activities as well as total phenolic content, increased with higher concentrations of MP, MPP, and PP, with MPP at 15 % showing the highest activity. All films exhibited over 50 % biodegradability within 15 days, suggesting their potential as sustainable, biodegradable edible packaging materials derived from tropical fruits (Susmitha, Sasikumar, Rajan, Padmakumar, & Nampoothiri, 2021b).

## 6 Conclusion

Mango byproducts (peels, seeds, leaves) have emerged as potent functional agents for enhancing biopolymer-based food packaging. Their integration addresses critical limitations of conventional plastics by imparting antioxidant, antimicrobial, and barrier functionalities while promoting biodegradability. MPE and pectin improve mechanical strength and UV protection, whereas seed starch (MKS) and kernel extracts enhance thermal stability and hydrophobicity. Leaf extracts contribute significant bioactivity and compatibility with diverse polymer matrices. These innovations extend the shelf life of perishable foods, mitigate agro-industrial waste, and reduce reliance on synthetic additives. The circular economy approach—transforming waste into high-value packaging materials—aligns with global sustainability goals, offering scalable solutions for the food industry.

## 7 Challenges and prospects

Despite the promising advancements highlighted in this review, several key challenges must be addressed to realize the full potential of mango byproduct-functionalized packaging. Foremost among these is overcoming the inherent variability in byproduct composition (influenced by cultivar, ripeness, and geography) through the development and adoption of standardized extraction protocols, particularly optimizing green techniques like microwave-assisted extraction (MAE) for scalable industrial production. Future research should aggressively pursue the creation of multifunctional systems, exploring synergistic combinations of mango-derived compounds (e.g., peel phenolics with seed lipids) within advanced hybrid matrices like nanocomposites and multilayer films; nanoencapsulation offers a promising avenue to enhance bioactive stability and enable controlled release. Concurrently, leveraging the natural pigments (e.g., carotenoids) in mango byproducts to develop pH-responsive colorimetric sensors presents a significant opportunity for advancing intelligent “smart” packaging capable of real-time freshness monitoring. Crucially, achieving regulatory approval and consumer acceptance necessitates comprehensive migration studies, cytotoxicity assessments, and sensory evaluations, alongside investigations into consumer perception of natural additives. Finally, quantifying the environmental benefits via rigorous life cycle assessments (LCA) comparing mango-waste films to conventional plastics is essential to validate and reinforce their ecological advantages. By systematically addressing these gaps—standardization, multifunctionality, intelligence, safety/acceptance, and environmental validation—mango-byproduct-functionalized films can successfully transition from promising lab-scale prototypes to mainstream, commercially viable applications, thereby driving significant sustainable innovation within the food packaging sector.

## CRediT authorship contribution statement

**Guihong Fang:** Writing – review & editing, Writing – original draft, Methodology, Investigation, Conceptualization. **Bangdi Liu:** Writing – review & editing, Writing – original draft, Supervision, Data curation, Conceptualization. **Junyan Guo:** Writing – original draft, Methodology. **Gulden Goksen:** Writing – original draft. **Mansuri M. Tosif:** Writing – original draft. **Shima Jafarzade:** Writing – original draft. **Parya Ezati:** Writing – original draft. **Ananthi Pandi:** Writing – original draft.

## Declaration of competing interest

The authors declare that they have no known competing financial interests or personal relationships that could have appeared to influence the work reported in this paper.

## Data Availability

No data was used for the research described in the article.
